# Effects of Individual Pre-Fledging Traits and Environmental Conditions on Return Patterns in Juvenile King Penguins

**DOI:** 10.1371/journal.pone.0020407

**Published:** 2011-06-08

**Authors:** Claire Saraux, Vincent A. Viblanc, Nicolas Hanuise, Yvon Le Maho, Céline Le Bohec

**Affiliations:** 1 Institut Pluridisciplinaire Hubert Curien, Université de Strasbourg, Strasbourg, France; 2 Centre National de la Recherche Scientifique, Unité Mixte de Recherche 7178, Strasbourg, France; 3 AgroParisTech ENGREF, Paris, France; 4 Centre for Ecological and Evolutionary Synthesis, Department of Biology, University of Oslo, Blindern, Norway; National Institute of Water & Atmospheric Research, New Zealand

## Abstract

Despite the importance of early life stages in individuals' life history and population dynamics, very few studies have focused on the constraints to which these juvenile traits are subjected. Based on 10 years of automatic monitoring of over 2500 individuals, we present the first study on the effects of environmental conditions and individual pre-fledging traits on the post-fledging return of non-banded king penguins to their natal colony. Juvenile king penguins returned exclusively within one of the three austral summers following their departure. A key finding is that return rates (range 68–87%) were much higher than previously assumed for this species, importantly meaning that juvenile survival is very close to that of adults. Such high figures suggest little juvenile dispersal, and selection occurring mostly prior to fledging in king penguins. Pre-fledging conditions had a strong quadratic impact on juvenile return rates. As expected, cohorts reared under very unfavourable years (as inferred by the breeding success of the colony) exhibited low return rates but surprisingly, so did those fledged under very favourable conditions. Juvenile sojourns away from the colony were shorter under warm conditions and subsequent return rates higher, suggesting a positive effect of climate warming. The longer the post-fledging trip (1, 2 or 3 years), the earlier in the summer birds returned to their natal colony and the longer they stayed before leaving for the winter journey. The presence of juveniles in the colony was more than twice the duration required for moulting purposes, yet none attempted breeding in the year of their first return. Juvenile presence in the colony may be important for acquiring knowledge on the social and physical colonial environment and may play an important part in the learning process of mating behaviour. Further studies are required to investigate its potential implications on other life-history traits such as recruitment age.

## Introduction

Population growth rate is a function of several life-history variables (juvenile and adult survival, age at maturity, breeding success, etc.), and fluctuations in only one of those parameters may have effects on the rate at which populations are growing or declining. Explaining and predicting population trends under various climate scenarios thus requires a thorough knowledge of species' life-history traits, which result from complex trade-offs between specific reproduction, growth and survival rates under particular environmental conditions [1]. Studies having considered these different life-history variables in an attempt to partition their contribution to population growth rate [2] (and references therein), have reached varied conclusions depending on species. However, due to methodological limitations, life-history traits relating to early life stages have been largely overlooked. While a growing body of literature relates early life stages to later life-history traits (see [3] as an example), most calculations of population growth rate through matrix models are still based only on adult survival and breeding success. Nonetheless, early life parameters are major components of life-history strategies, and capital factors shaping population dynamics (*e.g.*, in *Marmota flaviventris* [4]; in *Pygoscelis adeliae* [5]).

Recruitment into the breeding population has a critical impact on population turnover and population dynamics. In birds, however, the correlation between the number of young fledged by a population and that recruited into the same population is usually poor (median R^2^ = 0.25 from studies summarized in [6]). Thus, over the studied species, an average of as much as 75% of the variance in the number of recruits results from effects that occur between fledging and sexual maturity, and not from the number of fledglings produced. In seabirds, post-fledging return and survival are known to be affected by environmental conditions during the pre-fledging period [7]–[8], notably through several biological aspects including brood size, hatching date, and fledging mass ([9]–[12] and references therein). A number of studies have documented the crucial role of environmental factors (such as climate variability) on breeding success and chick survival. However, it remains unclear whether and how these factors have consequences on future life stages. After fledging, juveniles lack crucial life skills [13] and are exposed to high rates of predation [14]. Inexperienced juveniles typically exhibit a lower foraging efficiency compared to adults (reviewed in [13], [15]), as they undergo a learning period during which they acquire information on which feeding grounds are best and which hunting strategies are the most efficient. Their survival may accordingly be at stake ([3], [16]–[17] and references therein). Juvenile quality at fledging, which should reflect pre-fledging conditions, may then play an important role in juvenile survival and consequently, have strong impacts on population dynamics.

Variability in early life parameters should thus not be neglected when studying the population dynamics of a species. In particular, special attention should be given to early life parameters of top predators, which are used more and more as key indicators of environmental stress in various ecosystems (seabirds reviewed in [18]). Upper-level predators indeed integrate the effects of climate forcing throughout the food chain [19], and thus constitute good models for assessing ecosystem health. In this regards, king penguins (*Aptenodytes patagonicus*) provide a useful means for studying the impact of climate change [20]–[21], and although the species has been well studied [22]–[25], relatively little is known on the life-history traits of its early life stages. Juvenile penguins leave their colony as yearlings and become sexually mature at a minimum age of three or four years old but with an average age at first reproduction of six [23]–[24]. While they still need to come ashore for moulting, they do not have to return as often or stay as long in the colony as adults, the latter which, because of breeding activities, are central place foragers. Although early studies have stated that immature king penguins are seen again in their natal colony after a few years [23]–[24], how immature birds budget their time away from the colony yet remains poorly understood. Furthermore, previous studies relied on the monitoring of flipper-banded birds, and we know now the detrimental effects of flipper-bands on penguin fitness [21], [26].

Here, based on a 10-year automated transponder-based monitoring, we present the first study to consider the impacts of pre- and post-fledging environmental conditions, as well as the effect of individual parameters (i.e. sex, body condition and structural size) on the return rates of juvenile king penguins to their natal colony and lengths of their post-fledging trips away from the colony.

## Materials and methods

### Permits and ethics statement

All animals in this study were handled only once (during their first moult) in order to inject each individual with a subcutaneous transponder tag and to conduct morphological measurements. All procedures employed during this field work were approved by the Ethical Committee of the French Polar Institute (Institut Paul Emile Victor – IPEV) and conducted in accordance with its guidelines, also complying with French laws including those relating to conservation and welfare. Authorizations to enter the breeding site (permits n° 2005-191 issued on the 21^st^ of November 2005) and handle birds (permits n° 99/346/AUT issued on the 30^th^ of November 1999, 00/240/AUT issued on the 5^th^ of September 2000, 01/315/AUT issued on the 4^th^ of July 2001, 01/322/AUT issued on the 16^th^ of August 2001, 2003-113 and 2003-114 issued on the 7^th^ of October 2003, 2004-182 and 2004-183 issued on the 14^th^ of December 2004, and 2005-203 issued on the 1^st^ December 2005) were delivered first by the French “Ministère de l'Aménagement du Territoire et de l'Environnement” and then by the Terres Australes et Antarctiques Françaises (TAAF).

Handled animals were removed from the colony in order to minimize the disturbance to neighbouring birds and taken to a shelter a few meters away for manipulation. They were hooded to reduce their stress and manipulations lasted between 5 and 10 minutes. The transponder tags weigh 0.8 g and have no known adverse effects. They were shown not to affect survival of king penguins [27] or breeding success, recruitment or survival of tits [28]. Furthermore, concerns about infections should be minimal, as transponder tags were kept sealed sterile in iodine capsules (Betadine) and were removed from the capsules only by the process of injecting them into the bird. Moreover, Vétédine soap and alcoholic antiseptic solutions were used to disinfect the skin and the injecting needle before each insertion. Flesh wounds did not seem infected thereafter (personal observations on recaptured birds).

### Penguin monitoring

Our study was conducted on Possession Island (46°25′S, 51°45′E, in ‘La Grande Manchotière’ colony) in the Crozet Archipelago. From 1999 to 2005, 2509 10-month old chicks were randomly sampled during their moult, a few weeks before fledging and were implanted with passive transponder tags under the skin of their leg, without any other external mark. Mean tagging dates varied over years (range 12^th^ of November–9^th^ of December) due to annual differences in the timing of the moult period. A hundred birds were tagged later in the season in 2001 (January) and were thus discarded of the study to avoid the eventual bias of late fledging, leaving 2409 birds for the study. Each of our cohorts was considered representative of the year and was used to look at differences between years. The antennas buried under the usual and unique transit pathways in and out of the sub-colony allow for the continuous automatic collection of data on bird presence and movement. Although this automatic identification system [29] presents the major advantage of not requiring recapture and avoiding disturbance of the animals, it only concerns a part of the colony (ANTAVIA sub-colony, between 8 and 10 thousand breeding pairs, i.e. about one third of the colony). Thus, to obtain a complementary view, we also controlled for the presence or absence of juveniles in the rest of the colony by weekly visual observations (based on age dimorphism, such as beak colouration) and estimated their number.

We analysed detection data over 10 years, i.e. from early November 1999 to the end of May 2009. Considering the first five cohorts tagged between 1999 and 2003, nearly all chicks (i.e. 99.9%) which were seen again in the colony during this decade came back within one of the three years following their fledging departure (i.e. before May *n*+3). We thus included chicks tagged in 2004 and 2005 in this study, and then disposed of 7 cohorts. Birds which were never detected after tagging were considered to have either died in the colony before fledging or encountered a dysfunction of their tag and were thus discarded from the study (i.e. 34 animals discarded, leaving 2375 birds for the return behaviour study).

### Survey

Tagging year was defined as the year of reference (i.e. year *n*). After tagging, as chicks tended to frequently transit in and out of the sub-colony before leaving, we considered as departure date the last date at which the bird was automatically identified leaving the sub-colony during the austral summer of its tagging. Identically, we considered as return date the first date at which the bird was recorded back entering into the sub-colony. Duration on land before departure and trip duration were defined as the difference in days between departure date and tagging date, and between return date and departure date respectively. It is important to note that birds do not stay at sea during the whole post-fledging trip. Trip duration thus corresponds to the time spent away from the sub-colony and is composed of both time spent at sea and time spent on land outside the sub-colony. Finally, return rate was defined as the ratio of the number of birds detected again after their fledging (in one of the three following years) over the number of birds that left the colony.

Additionally, the automatic identification system allowed us to monitor the activities of the birds after their first return in order to determine the time spent in the natal sub-colony and see whether they attempted breeding. However, as birds need to frequently resume foraging trips to feed themselves, investigating their activity and use of the colony was only possible by considering the whole period during which they regularly visited the colony (frequency of visits >1 per month), thus including periods of time when birds were physically present in the sub-colony and periods when they were out. The birds were considered as attempting to breed when at least two incubation shifts were observed, meaning that an egg was laid and incubation had started.

### Individual traits: sex, structural size and body condition

Birds tagged after 2000 were blood-sampled at tagging and sexed using microsatellite DNA-analyses (adapted from [30]). In the absence of DNA-samples, i.e. for the first cohort, gender was determined by analysing the chronology of the sex-specific incubating shifts of their following breeding cycles [22], [25].

For each bird, flipper and beak lengths were measured at tagging [22]. These two morphologic measurements are good descriptors of king penguin structural size and are highly repeatable measurements [31]. As beak and flipper lengths were correlated (Spearman's rank correlation, P<0.001, r = 0.41, n = 2509), we used a principal component analysis to establish an index of structural size (SSI) as follows: SSI = PC1 = 0.26 * Beak+0.96 * Flipper. The first principal component (PC1) between these two parameters explained 84% of the variation.

Body mass is highly variable in king penguins and can be associated with differences in nutritional status as well as structural size. Differences between body mass and structural size thus constitute a good index of nutritional state [32]. Body condition was then defined as the residuals of a regression of body mass on SSI ([32]; R^2^ = 0.11, P<0.001). As birds were tagged at a comparable moulting stage, BC at tagging was considered as a valid indicator of bird quality and was used without further correction.

As departure dates and BC were correlated (Spearman's rank correlation test; r = −0.31, P<0.001, N = 2473), the impact of both variables on return rates or dates was studied using BC and the residuals of BC on departure dates as input variables in our models.

### Environmental conditions

Environmental conditions have been shown to affect population dynamics at both local and global spatial scales [33]. The use of ‘weather packages’ and large-scale climate indexes (global indices encompassing a combination of weather features, see [34]), such as the Southern Oscillation Index (SOI), are good candidates for explaining the effects of environmental variability on top-predators of the Southern Ocean, such as penguins [35]. Negative SOI values indicate El-Niño events, whereas positive values indicate La Niña events [36]. Monthly SOI (calculated from the monthly fluctuation in the air pressure difference between Tahiti and Darwin) were obtained from the Australian Bureau of Meteorology.

Since changes in Sea Surface Temperature (SST) have repercussions on the primary production and the food chain [37], SST is frequently used as a local proxy of abundance and distribution of prey for king penguins [35]. Daily SST values (in °C) were obtained from the National Ocean and Atmospheric Administration. However, little is known on the location of feeding grounds in sub-adult king penguins. They may exhibit a similar behaviour as the one of the adults that either forage around the Polar Front (PF) or the Marginal Ice Zone (MIZ), depending on the season [38]. However, unlike breeders, juveniles are not central place foragers. This could have strong impacts on the location of their feeding grounds. For instance, some sub-adult birds, probably originating from Macquarie Island, have been spotted in Australia or New-Zealand [23], which hints to the fact that they could well go as far up north as the subtropical area. We therefore tested for SST averaged on different areas to investigate the effect of temperature on post-fledging trips. A global area from the sub-tropical front to the MIZ (38–60°S, 46–56°E) was tested and divided in four small sub-areas surrounding notable oceanographic structures (38–42°S around the sub-tropical front, 42–46°S around the sub-Antarctic front, 48–52°S around the PF, 56–60°S around the MIZ). Oceanic fronts and areas associated with the seasonal sea ice retreat are indeed very productive regions [39] and important foraging grounds for top-predators [40].

Environmental conditions at sea were assessed over several periods. We considered mean values during the entire post-fledging trip, the first two months, the first year, or the first winter (May–September) spent outside the sub-colony, and finally during the two last months preceding juvenile return at the colony.

The breeding success of the colony (Le Bohec *et al.* in prep.) was also used as a proxy for the conditions endured during the rearing period. Years of high breeding success (such as 2002 or 2004) could thus be viewed as more favourable years, compared to years of lower breeding success.

### Statistics

All statistics were computed using R v. 2.9.0. and SPSS v. 17.0. statistical softwares. Data were analysed using a maximum of likelihood generalized linear model approach. Generalized linear models were fitted with either Poisson distribution concerning trip duration or binomial distribution concerning return rate. Model selection was based on Akaike's Information Criterion (AIC) study, using both ΔAIC and AIC weights. In general, the model exhibiting the lowest AIC was selected, except when ΔAIC<2. In that specific case, AIC weights were examined as well as the number of parameters (models with smaller number of variables being favoured to avoid overparametrization, i.e. the most parsimonious models). The explained deviance of the model (in relation to the null model, i.e. the relative variability explained by the model compared to the entire variability in the dataset) and p-values were then used to conclude as to the effect of the parameters.

Some birds only returned to the colony after several years. Therefore, to explain the three-state categorical variable return year (distribution of birds in different yearly return groups), we computed ordinal logistic regressions, using the lrm function of the ‘Design’ package in R. Using Harrell's recommendation of graphical method, the parallel slopes' assumption was verified, validating the use of ordinal logistic regression [41]. To investigate the effect of environmental conditions at sea on the proportion of birds within the three years of return, we also defined two different ratios for each cohort: i) *ratio1* corresponded to the number of birds coming back in year *n*+1 over the number of birds coming back at the colony overall years, and ii) *ratio2* corresponded to the number of birds coming back in year *n*+2 over the number of birds coming back on years *n*+2 and *n*+3. Then we used the SOI averaged on the first year at sea to explain the decision of coming back or not after this year (*ratio1*) and the SOI averaged on the two first years to explain the decision of coming back or not after two years (*ratio2*). We pooled *ratio1* and *ratio2* together in *ratio* after standardisation (to avoid an offset difference in between the two groups) and ran a single model with SOI as an explanative variable of *ratio*.

In order to compare different groups (e.g., males versus females, or in between cohorts), we first checked for normality and homoscedasticity between groups, and non-parametric tests were used consequently (including Wilcoxon rank-sum test and Mood median test). Variables were considered significant for P<0.05 and Bonferroni's correction was applied whenever multiple comparisons were tested (differences were thus considered significant for P<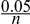 with *n* the number of comparisons done).

## Results

### Summer of departure

**Figure 1:**
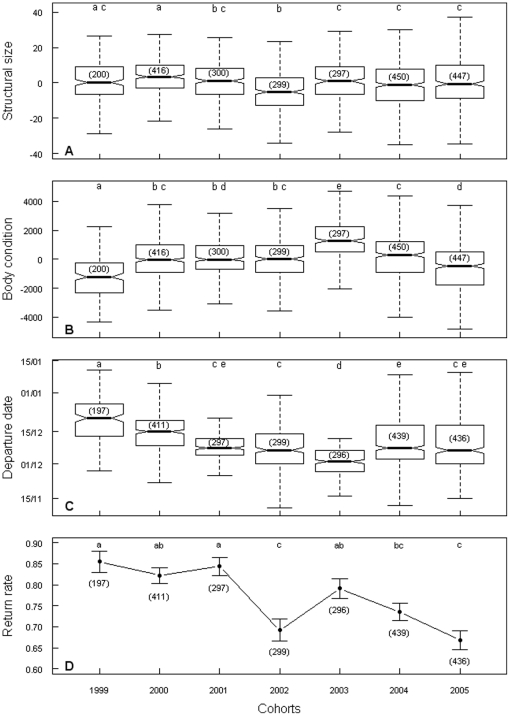
Inter-cohort differences in a) structural size (SSI), b) body condition (BC), c) departure date and d) return rate. Sample size is indicated in brackets. Values not sharing a common letter are significantly different for P<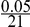 according to pairwise Bonferroni adjusted Mood tests. Panels a to c represent boxplots, while panel d shows means ± SE.

The sex ratio of our sample was almost balanced between sexes (52% of males *vs.* 48% of females, P = 0.13). Structural size indexes (SSI) were relatively similar between cohorts, with only two cohorts standing apart (cohorts 2000 and 2002, Fig. 1a). Body condition (BC) on the other hand was highly different between cohorts (Fig. 1b). Juvenile king penguins all fledged during austral summer. However, departures stretched over a long period (i.e. almost 5 months), extending from 9^th^ of November until 22^nd^ of March (Fig. 1c).

Sex, BC, SSI and cohort were used to explain differences in duration before departure. The model with all four variables was retained as best model by AIC selection (AIC = 17662, Explained deviance = 35%, ΔAIC = 119 with the closest model, i.e. model without sex) and all variables were significant (all P<0.001). However sex accounted for less than one percent in overall dispersal. Birds of better BC left earlier, whereas birds of greater size stayed longer.

### Return rates

The global return rate obtained was of 77%, i.e. 1838 returned birds out of 2375 leaving the colony (all 7 cohorts over the whole period). Return rates varied significantly between cohorts ranging from 68% for the 2005 cohort, to 87% for the 1999 cohort (Fig. 1d).

**Figure 2:**
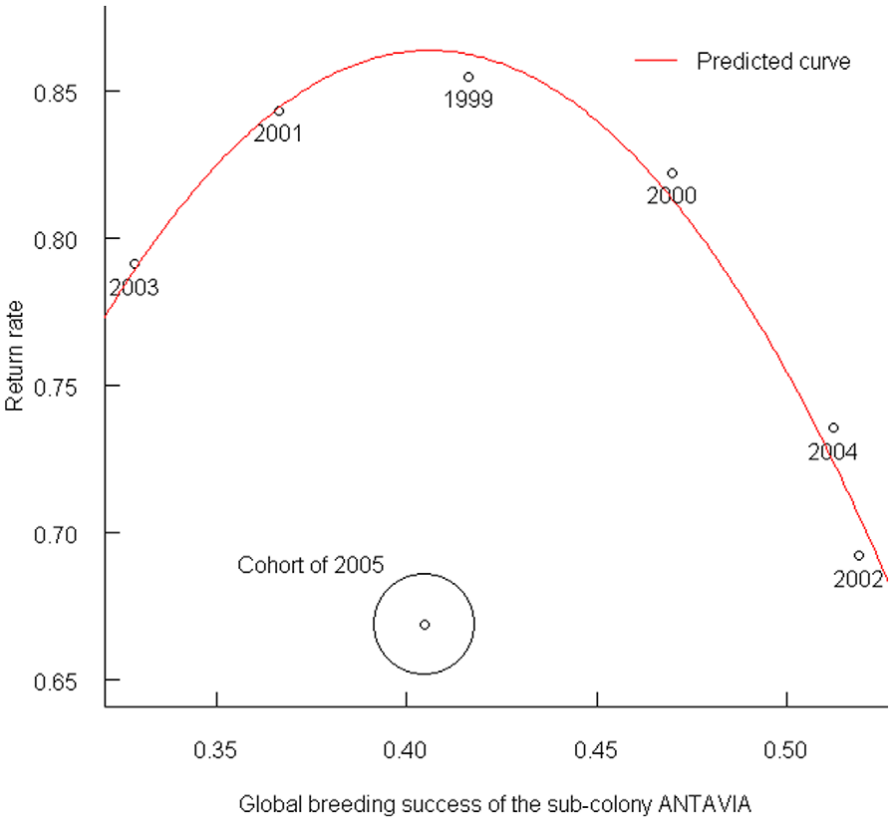
Mean return rate per cohort related to the global breeding success of the colony. Fitted curve of the linear regression Return rate ∼BS+BS^2^ without the 2005 cohort is indicated in red.

Plotting the return rates of these seven cohorts against population breeding success (BS), i.e. a proxy for the conditions endured during the rearing period, highlighted a potential quadratic effect of environmental conditions prior fledging on these return rates except for the 2005 cohort (Fig. 2). There were no significant effects of either BS or BS^2^, when running the model on all seven cohorts. However, excluding the 2005 data, we found an almost perfect fit between those variables (Return rate ∼BS+BS^2^, P = 0.004 and 0.003 respectively, n = 6, R^2^ = 0.98; Fig. 2).

We also found an effect of climate at sea (of both SOI and SST regardless of the area over which it was averaged on) on individual return probability. Model selection showed that SOI averaged on the whole trip and SST averaged on the whole trip and on the northern area (38–42°S, around the sub-tropical front) were the best explicative climatic variables (Table 1, models R1 to R10). Adding biological variables, model R1.3 appeared as the minimal adequate model (Explained deviance = 25%, AIC = 1929, k = 10, N = 2375, Table 1, models R1 to R1.6), which predicted that return rate was affected by climate, BC, sex and year of fledging. Birds in better condition were more likely to return to the colony (P<0.001), while warmer conditions (higher SST and lower SOI) had a positive effect on the return rate of sub-adult king penguins (both P<0.001). On average, males presented a higher return rate than females (78% *vs.* 75%), but this varied substantially between years, from 15 percentage points more for males in 2005 (74% *vs.* 59%) to 7 percentage points more for females in 2003 (83% *vs.* 76%).

**Table 1:** Model selection to explain individual return rate variability in juvenile king penguins.

N°	Animal characteristics	Year	Depart	Climatic variables	AIC	ΔAIC	w_i_	k	ED
**R1**				**SOI_w_+SST_w,z1_**	**2119.6**	**0**	**1**	**2**	**17%**
R2				SOI_w_+SST_w,z2_	2258	138.4	<0.001	2	11%
R3				SOI_w_+SST_w,z3_	2345	225.4	<0.001	2	11%
R4				SOI_w_+SST_w,z4_	2315.8	196.2	<0.001	2	8%
R5				SOI_w_+SST_w,tot_	2260.6	141	<0.001	2	9%
R6				SOI_w_+SST_2m,z1_	2528	408.4	<0.001	2	<1%
R7				SOI_w_+SST_y1,z1_	2497.1	377.5	<0.001	2	<1%
R8				SOI_w_+SST_wint1,z1_	2528.6	409	<0.001	2	<1%
R9				SOI_w_	2529.7	410.1	<0.001	1	<1%
R10				SST_w,z1_	2405.5	285.9	<0.001	1	6%
R1				SOI_w_+SST_w,z1_	2119.6	191	<0.001	2	11%
R1.1	BC+SSI+SEX	Year	Depart	SOI_w_+SST_w,z1_	1929.9	1.3	0.22	12	25%
R1.2	BC+SSI+SEX	Year		SOI_w_+SST_w,z1_	1929.3	0.7	0.30	11	25%
**R1.3**	**BC+SEX**	**Year**		**SOI_w_+SST_w z1_**	**1928.6**	**0**	**0.43**	**10**	**25%**
R1.4	BC+SEX			SOI_w_+SST_w,z1_	2014.3	85.7	<0.001	4	21%
R1.5	BC	Year		SOI_w_+SST_w,z1_	1998.1	69.5	<0.001	9	22%
R1.6	SEX	Year		SOI_w_+SST_w,z1_	1933.4	4.8	0.04	9	25%

Best models are indicated in bold. ΔAIC is the difference of AIC compared to the best model. w_i_ corresponds to the AIC weight and represents the probability of this model being the best among the models presented. k is the number of parameters in the model. ED stands for explained deviance and has been calculated as the ratio of the deviance explained by the model (null deviance – residual deviance) on the null deviance.

BC and SSI are the body condition and structural size of the animal before departure. Depart is the residual of BC on the date of departure of the bird. SOI_w_ and SST_w_ are the Southern Oscillation Index and Sea Surface Temperature averaged on the whole trip for birds having returned and on the 3 years following the departure for those never seen again. SOI_y1_ was the average of SOI on the first year following departure. SST_2m_, SST_wint1_, SST_y1_ were averaged on the first 2 months, the first winter and the first year.

SST was averaged on different areas, z1 to z4 corresponding to areas surrounding the different fronts from north to south: z1, sub-tropical front; z2, sub-antarctic front; z3, polar front; z4, marginal ice zone and tot being the whole area from north bounding of z1 to south bounding of z4.

### Return dates

**Figure 3:**
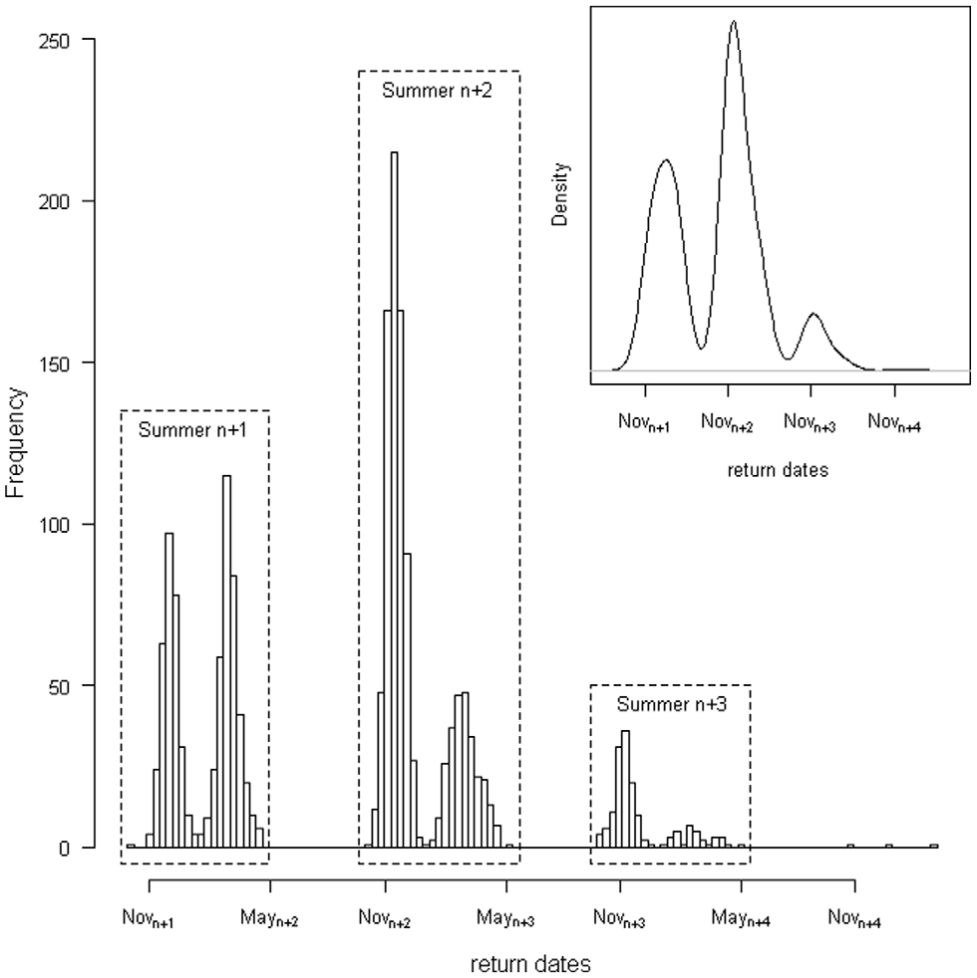
Return date of post-fledging king penguins after their first trip out of the sub-colony (density and histogram).

The first returns to the colony were observed occurring in three distinct periods during each of the three austral summers following juvenile fledging, regardless of the cohort (upper-right panel of Fig. 3). Overall, the second return summer was far greater than the other two, i.e. 37% of the birds returned after a year, 54% after two, and only 8% after three. No birds were recorded returning during the austral winter. These results were confirmed by weekly observations of the whole colony during the 7-year study period (only one sub-adult was seen during winters of 2000 and 2001 in the whole colony). Other than during these three summers, we detected only three penguins returning to the colony, all of them arriving during the austral summer of year n+4.

Each summer was also composed of two return peaks. Hereafter, we refer to the three return years (n+1, n+2 or n+3) as ‘return year’, whereas the yearly peaks are referred to as ‘peak’. The more years sub-adults stayed away from their sub-colony, the earlier in the season they made their first returns to the sub-colony. Return year n+1 was indeed composed by two very similar peaks (46% in the first peak *vs.* 54% in the second peak), whereas return years n+2 and n+3 presented unbalanced ratios with 73% and 79% of returns in the first peak respectively. Moreover, peaks of year n+3 occurred earlier than peaks of year n+2, which themselves occurred earlier than peaks in year n+1 (Fig. 3, median days of the two peaks 2^nd^ of December/27^th^ of February *vs.* 16^th^ of November/25^th^ of February *vs.* 8^th^ of November/21^st^ of February for return year n+1, n+2, n+3, respectively).

### Sea trip duration

Trip duration of birds was significantly different between cohorts (Kruskal-wallis test, P<0.001). Birds of the 2005 cohort spent significantly more time away from their sub-colony than any other cohort (Pairwise Wilcoxon rank-sum tests adjusted with Bonferroni correction: P<0.001 for the 2005 cohort *vs.* every other cohort).

A difference in the mean trip duration between cohorts could be the consequence of two different situations: 1- the proportion of birds between the 3 years of return is different between cohorts (*ratio*) 2- the proportion is the same, but durations are not the same inside a single year of return. SOI negatively affected *ratio* (P = 0.05), suggesting that in warmer conditions (low SOI), the proportion of birds coming back early increased. As for individual parameters, sex and BC had no effect on the probability to come back in one of the three years. The best selected ordinal logistic regression indicated that residuals of departure on BC had a positive effect on return year (P = 0.005), i.e. that, independently of BC, those birds which left the colony later, also spent a longer period away from their sub-colony. SSI had a negative effect indicating that smaller birds had a higher probability of coming back in years n+2 or n+3 than in year n+1 (P = 0.05). Finally cohorts also had a significant effect (P<0.001) and differences between cohorts were asserted using Bonferroni corrected Wilcoxon rank-sum tests (see Fig. S1).

As for distribution in peaks inside return years, the best model (model P1.3, Explained deviance = 96%, AIC = 108.4, k = 9, N = 1902; Table S1) predicted that it was almost entirely explained by SOI averaged on the last year and the global area SST averaged on the last 2 months before return (both P<0.001). SOI had a positive effect and SST a negative effect, indicating that under warm conditions, birds returned earlier, i.e. in peak one instead of peak two. Birds of smaller SSI at fledging might tend to return later (P = 0.01), even if size added only little information (ΔAIC = 0.6).

### Post-return activity

**Figure 4:**
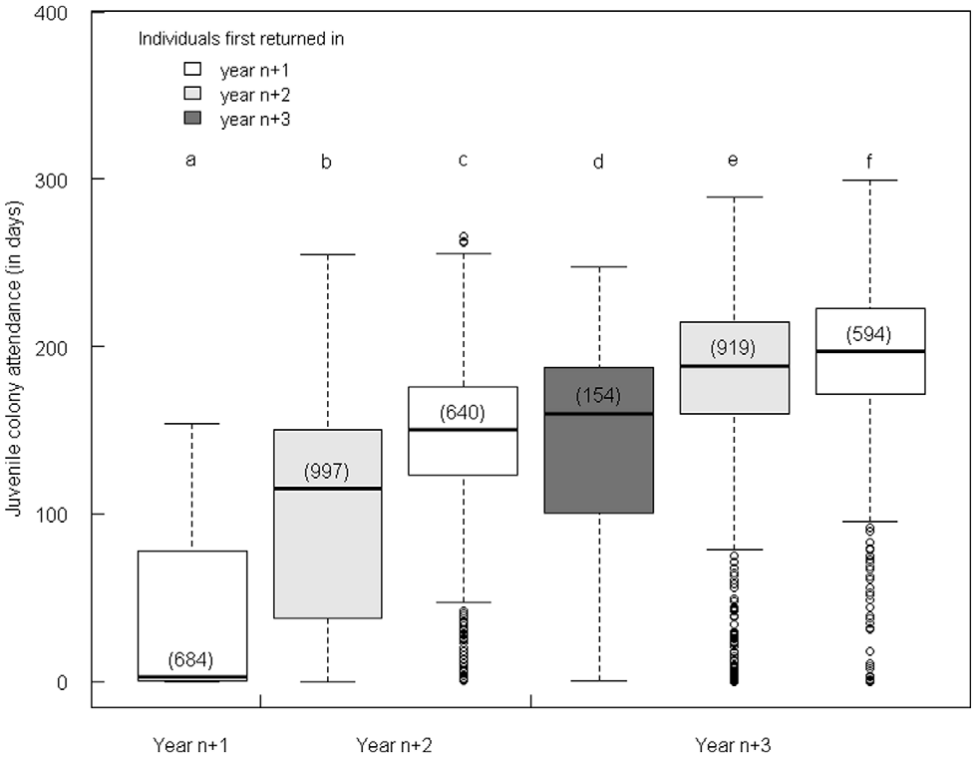
Colony attendance (in days) upon return in the colony in the three years following their departure depending on the year of first return. Values not sharing a common letter are significantly different for P<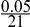 according to pairwise Bonferroni adjusted Mood tests. Median ± SE: 2±2 days, 115±2 days, and 160±5 days spent upon return for birds first returning after 1, 2 and 3 years respectively.

Weekly observations of the whole colony all along the ten years of study allowed us to determine that the period of moult for the sub-adults extended from mid-November to the end of January. Upon their first return, juvenile birds continued to visit the colony for an average of 79 days (more than 2 ½ months), ranging from 0 to 255 days (about 8 ½ months). Independently of their year of return, the birds arriving at the beginning of the summer (i.e. in the first of the two peaks of each summer) visited the colony during a significantly longer period than the birds arriving late (median ± SE: 124±2 days *vs.* 3±1 days, P<0.001). In addition, the longer they stayed away from their sub-colony during their post-fledging trip, the more they attended the colony on their return (Fig. 4). More than half of the birds coming back on the first year attended the colony for less than a week (i.e. 56%) compared to only 16% for birds first returning after two years, and 4% for birds first returning after three. Furthermore, almost all birds returning to the colony in one of the two first years returned again on the following summers, provided that they did not die (99.6% from n+1 to n+2 and 99.3% from year n+2 to n+3). When coming back for the second time, birds spent more time at the colony than birds of the same age coming back for the first time (Fig. 4). Identically, in year n+3, birds coming back for the third time at the colony spent significantly more time than birds coming back for the second time (Fig. 4).

Although a few birds attempted to breed upon their first return to the colony (1.5%, i.e., 28 over 1835 birds), all failed in fledging a chick. The proportion of breeders increased with age at first return (only 0.1% *vs.* 1.8% *vs.* 5.7%, for birds coming back in year n+1, n+2 and n+3 respectively). 61% of these birds trying to reproduce upon their first return to their natal sub-colony were females, in spite of the higher number of males studied (2.2% of females engaged in reproduction *vs.* 1.3% of males).

## Discussion

### Return rate, survival, emigration

In free-living non-banded king penguins, we found that more than ¾ of the fledglings return to their natal population after their first sojourn at sea. Over 7 consecutive years and for 2375 penguins, post-fledging return rates to the natal sub-colony ranged from 68% to 87% depending on cohort (average 77%). This proportion is far greater than has been previously found (i.e. 5.6 to 39% [24]). Moreover, our return rates might even underestimate survival as some of those birds which were not detected again in the natal sub-colony, might have either emigrated or established themselves in another sub-colony of the same population. Emigration is usually thought to be very low in adult king penguins (94% of fidelity to breeding site [23]). However, when compared to adults, the higher proportions of juveniles seen in other colonies (see [23], and 1.5% *vs.* 0.4% in [24]) suggest that juveniles might come on land out of their natal colony more often than adults. Yet, our data suggest that juvenile dispersal might be small in this colony, and global return rate may be a good estimator of survival.

Survival is usually much lower for juveniles than for adults [6], [42]. Explanations are 1- the ‘constraint hypothesis’, suggesting a lack of experience among juveniles for different activities such as foraging, avoidance of predators, etc. [42] and 2- the ‘selection hypothesis’, stating that birds with less adapted phenotypes disappear in early stages of life and thus that older population categories are only composed of good phenotypes [6], [42]. Unlike a lot of birds, king penguins exhibit a very low breeding success and fledglings have already overcome a strong selective pressure. Since we found here that more than 70% of the fledglings returned to their natal colony and were still alive three years after fledging, i.e. an average annual return rate of about 90%, we suggest that selective mechanisms for juvenile king penguins should mostly operate before fledging. Little is known regarding the ability to forage in king penguin juveniles. Yet, if there is an effect of age and experience on foraging (as in many birds [15], and even other penguin species [43]), lower juvenile foraging skills [13] might not be a strong limiting factor for survival in king penguins. Indeed, a lower efficiency of juveniles could be compensated for by longer periods of foraging since they are not subjected to the same constraints as breeding adults.

### Body condition and structural size: departure and return

Chicks in poorer condition at fledging left the colony later, suggesting either that it took them longer to complete their moult (a very energetic process) or that, being too weak to leave, they were compelled to stay longer begging for food before departing. A minimal energy capital may then be required to depart at fledgling. Yet, chicks do not depart from the colony with a maximal body condition (BC); rather, they go on fasting and lose weight before leaving, which probably improves their ability to perform prolonged immersion and deep diving [44]. Chicks of smaller structural size at fledging (SSI) left the colony earlier than bigger ones. According to allometric equations and surface to volume ratios, smaller birds should see their energy reserves depleted more rapidly than bigger ones (due to higher specific metabolic rates [45]) and may thus reach this optimal body condition after a shorter time of energy depletion.

BC at departure had a significant positive impact on return rate but no effect on trip duration. The opposite trend was observed for SSI, which did not affect return rate but had a negative effect on trip duration. BC is a good index of energetic reserves and can be critical for survival [46]. A positive correlation between body mass and juvenile survival (directional selection [47]) has indeed been highlighted in mammals [48] as well as in birds [14], [46], [49]–[51]. In king penguins, BC at departure presumably has a strong impact during the period spent to reach the first feeding grounds (and consequently survival at that time) but unlikely so on the duration of the entire trip (which lasts for more than a year, more time than needed to rebuild BC). Greater SSI however, could be an inherent advantage for juvenile survival in king penguins, as shown in other species [50]. Different explanations have been advanced, from inter-individual differences in anti-predator capacities [17] or inter-individual competitive capacities [11], [49], to differences in foraging efficiency [52]. If the effect of SSI is not critical enough to negatively affect global return rate in juvenile king penguins, we suggest that birds with bigger flippers might be more efficient in swimming, diving and foraging (as has been found in seals [52]), resulting in a shorter time to return to the colony. Larger body size may also confer the advantages of lower mass-specific metabolic rate according to allometric equations [45]. Such a lower metabolic rate may then increase efficiency at converting acquired resources into fat reserves [53]. Larger birds would therefore be able to acquire earlier a sufficient body condition to return to the colony, where penguins endure obligate fasting.

### Inter-annual variations and climate

The high variability observed in the global return rate and duration spent at sea between cohorts of juvenile king penguins may be a consequence of varying environmental conditions, either prior to fledging or during the post-fledging period spent at sea. Indeed, conditions experienced early in life may have important consequences on individual fitness [54]. For instance, individuals born during years of low food availability will present low phenotypic quality, leading to high subsequent juvenile mortality. In this study, juveniles fledged under unfavourable conditions indeed exhibited low return rates. However, return rates did not increase linearly with favourable conditions, suggesting that an opposite mechanism occurred. Selection mechanisms happen at different life stages and the ‘selection hypothesis’ stating that birds with less adapted phenotypes would disappear in early stages of life could occur more or less early depending on the environment [55]. In common terns, under harsh conditions, most weak individuals are already eliminated prior to fledging, whereas in favourable years, many juveniles of lower quality survive the pre-fledging period but may die later when environmental constraints become critical [55]. Similarly, king penguin chicks fledged in years of very favourable conditions may be of highly heterogeneous quality as a result of low selection pressure in these years, and thus present lower return rates than birds fledged under ‘normal’ conditions. An alternative explanation could be a condition-dependent dispersal. Indeed, one can imagine that under favourable conditions, king penguin juveniles would have a higher ability to disperse. However, according to the concept of “voting with their feet”, we could expect the opposite, with higher dispersal when conditions are poor (decreasing breeding success has for instance been shown to increase dispersal rate in seabirds, [56]–[57]).

Interestingly, those birds fledged in 2005 presented an especially low return rate, not attributable to delayed returns, as no birds were observed in the colony after the three usual return years. However, these birds were reared after the December 2004 tsunami, which greatly affected the studied colony despite being located some 6500 km away from the epicentre [58]. Breeding success was not directly impacted by flooding within the studied sub-colony, as it is away from the shores. Nonetheless, chick-rearing was harsh for the breeders, due to high levels of stress and aggressiveness throughout the colony. In addition, physical disturbances such as tsunamis are considered to be important factors structuring marine communities [59] (i.e. biotic communities, physical habitats and nutrient distribution) and exploitable resources could thus be significantly disturbed [60]–[61]. As long-lived seabirds, king penguins are expected to invest a fixed amount in current reproduction and offspring therefore to support the whole cost of environmental conditions [62]. Consequently, chicks reared after the tsunami were presumably of low quality (this cohort indeed had a very low mean BC at fledging), explaining their poor post-fledging return rate. Further, prey distribution may still have been disturbed when chicks fledged, because of inertia in the ecosystem delaying the return to a new steady state. Survival right after fledging could thus have been strongly impacted.

Finally, under warmer conditions, juveniles survived better and returned earlier. According to adult survival trends (decreased survival with warm temperatures in their foraging grounds during winter [35]), we would have expected the opposite result. However, juveniles and adults may display differences in foraging, related either to experience or different needs. For instance, nutritional requirements may be different, as juveniles may need higher levels of protein to finish their growth [63] or conversely less energetic prey, as they only forage for themselves [64]. Furthermore, juveniles may also forage at different locations since they do not have the constraints of central place foragers as breeding birds do. Unlike breeding adults, which mostly forage in two specific regions [38], juveniles are thus free to go and forage wherever they need to. Barrat [23] suggested that some juveniles could go as far up north as the subtropical area. In our study, we found as best explanatory climatic variable the SST averaged around the subtropical front, suggesting that this area may play a role for juvenile king penguins. The use of tracking methods (such as satellite tracking or GLS) or stable isotopes could then be valuable options to acquire knowledge on their feeding locations.

### Benefits of early returns

Average age at first breeding in king penguins is reported to be 6 years old [24], however sexual maturity is probably reached earlier (around 3) as some birds have been seen to attempt breeding at 3 or 4 ([23], personal observations). Importantly, we show here that birds are coming back in one of the three summers following their departure, i.e. between age 2 and 4, with as much as more than 90% coming back at 2 or 3. Moreover, all returns, without any exception, were recorded during the austral summer (from November to May). The return peaks of juvenile king penguins thus coincided with the breeding period, yet only a few of them attempted breeding. A possible explanation could be that juveniles need to return for moulting, which coincides with breeding. Based on weekly observations of the whole colony, we determined that their moult ranges between mid-November and the end of January. However, two different peaks of returns were observed in each year, the second peak occurring at the end of February. Only birds returning in the first peak could thus have come for moulting purposes; however, those spent far more time than required for moulting. Therefore, young king penguins do not return to their natal colony exclusively for moulting purposes. We suggest that they engage in courting but are not selected as preferred mates by their conspecifics, thus failing to breed. Pairing is indeed highly competitive in king penguins and we may assume that young birds are at a disadvantage. In particular, older birds are known to present stronger secondary sexual characters, such as conspicuous ornamental colours of both beak and plumage [65]. In our study, older juveniles spent more time at the sub-colony, suggesting that the older they are when they arrive at the colony, the more they try to engage into breeding. Furthermore, birds coming back for the first time as very young individuals (i.e. at age two), later spend significantly more time in the sub-colony during the subsequent summers (at ages three and four) than other birds of the same age, i.e. three or four, coming back for the first time. If, as suggested by Barrat [23], their presence at the colony is an important part of the establishment of reproductive behaviour, birds returning earlier in life would be able to gain more experience and better knowledge of their reproductive site (this includes best locations in the colony, avoidance of predators, or/and any social knowledge such as potential mates, brood neighbours, etc.). Since, however, few birds come back at age two; this strategy probably incurs other costs such as risks linked to the aggressive behaviour of breeders. Further studies relating breeding parameters such as recruitment age and age at first breeding success with age at first return to the natal group may help in answering this question.

## Supporting Information

Figure S1 **Distribution of the returns of sub-adult king penguins among the 3 years of returns depending on cohorts.** Values not sharing a common letter are significantly different.
(TIFF)

Table S1 **Competitive models tested to explain peak of return inside a return year.**
(DOC)

## References

[1] Stearns SC (1989). Trade-offs in life-history evolution.. Functional Ecology.

[2] Oli MK, Dobson FS (2003). The relative importance of life-history variables to population growth rate in mammals: Cole's prediction revisited.. American Naturalist.

[3] Grüebler MU, Naef-Daenzer B (2010). Fitness consequences of timing of breeding in birds: date effects in the course of a reproductive episode.. Journal of Avian Biology.

[4] Oli MK, Armitage KB (2004). Yellow-bellied marmot population dynamics: demographic mechanism of growth and decline.. Ecology.

[5] Wilson PR, Ainley DG, Nur N, Jacobs SS, Barton KJ (2001). Adélie penguin population change in the pacific sector of Antarctica: relation to sea-ice extent and the Antarctic Circumpolar Current.. Marine Ecology Progress Series.

[6] Newton I (1989). Lifetime Reproduction in Birds.

[7] Hedgren S (1981). Effects of fledging weight and time of fledging on survival of guillemot Uria aalge chicks.. Ornis Scandinavica.

[8] Harris MP, Frederiksen M, Wanless S (2007). Within- and between year variation in the juvenile survival of common guillemots Uria aalge.. Ibis.

[9] Perrins CM, Harris MP, Britton CK (1973). Survival of young Manx shearwaters Puffinus puffinus.. Ibis.

[10] Jarvis MJF (1974). The ecological significance of clutch size in the South African gannet Sula capensis (Lichtenstein).. Journal of Animal Ecology.

[11] Spear L, Nur N (1994). Brood size, hatching order and hatching date: effects on four life-history stages from hatching to recruitment in western gulls.. Journal of Animal Ecology.

[12] Cam E, Monnat J-Y, Hines JE (2003). Long-term fitness consequences of early conditions in the kittiwake.. Journal of Animal Ecology.

[13] Marchetti K, Price T (1989). Differences in the foraging of juvenile and adult birds: the importance of developmental constraints.. Biological Review.

[14] Naef-Daenzer B, Nuber W (2001). Differential post-fledging survival of great and coal tits in relation to their condition and fledging date.. Journal of Animal Ecology.

[15] Wunderle J (1991). Age-specific foraging proficiency in birds.. Current Ornithology.

[16] Lack DL (1954). The natural regulation of animal numbers.

[17] Sullivan K (1989). Predation and starvation: age-specific mortality in juvenile juncos (Junco Phaenotus).. Journal of Animal Ecology.

[18] Durant JM, Hjermann DØ, Frederiksen M, Charrassin JB, Le Maho Y (2009). The pros and cons of using seabirds as ecological indicators.. Climate Research.

[19] Boyd IL, Murray A (2001). Monitoring a marine ecosystem using responses of upper trophic level predators.. Journal of Animal Ecology.

[20] Le Maho Y, Gendner J-P, Challet E, Bost C-A, Gilles J (1993). Undisturbed penguins as indicators of changes in marine resources.. Marine Ecology Progress Series.

[21] Saraux C, Le Bohec C, Durant J, Viblanc VA, Gauthier-Clerc M (2011). Reliability of flipper-banded penguins as indicators of climate change.. Nature.

[22] Stonehouse B (1960). The king penguin Aptenodytes patagonica of South Georgia. I. Breeding behaviour and development. Falkland Islands Dependencies Survey.. Scientific report.

[23] Barrat A (1976). Quelques aspects de la biologie et de l'écologie du Manchot Royal (*Aptenodytes patagonicus*) des îles Crozet.. Comité National Français de la Recherche Antarctique.

[24] Weimerskirch H, Stahl JC, Jouventin P (1992). The breeding biology and population-dynamics of King Penguins Aptenodytes patagonica on the Crozet Islands.. Ibis.

[25] Descamps S, Gauthier-Clerc M, Gendner JP, Le Maho Y (2002). The annual breeding cycle of unbanded king penguins Aptenodytes patagonicus on Possession Island (Crozet).. Avian Science.

[26] Gauthier-Clerc M, Gendner JP, Ribic CA, Fraser WA, Woehler E J (2004). Long-term effects of flipper-bands on penguins.. Proc R Soc Lond B.

[27] Froget G, Gauthier-Clerc M, Le Maho Y, Handrich Y (1998). Is penguin banding harmless?. Polar Biol.

[28] Nicolaus M, Bouwman K, Dingemanse N (2009). Effect of PIT tags on the survival and recruitment of Great Tits.. Ardea.

[29] Gendner JP, Gauthier-Clerc M, Le Bohec C, Descamps S, Le Maho Y (2005). A new application for transponders in studying of penguins.. Journal of Field Ornithology.

[30] Griffiths R, Double MC, Orr K, Dawson RJG (1998). A DNA test to sex most birds.. Molecular Ecology.

[31] Fahlman A, Halsey LG, Butler PJ, Jones DR, Schmidt A (2006). Accounting for body condition improves allometric estimates of resting metabolic rates in fasting king penguins, Aptenodytes patagonicus.. Polar Biology.

[32] Schulte-Hostedde A, Zinner B, Millar JS, Hickling G (2005). Restitution of mass-size residuals: validating body condition indices.. Ecology.

[33] Stenseth NC, Mysterud A, Ottersen G, Hurrel JW, Chan KS (2002). Ecological effects of climate fluctuations.. Science.

[34] Stenseth NC, Mysterud A (2005). Weather packages: finding the right scale and composition of climate in ecology.. Journal of Animal Ecology.

[35] Le Bohec C, Durant J, Gauthier-Clerc M, Stenseth NC, Park YH (2008). King penguin population threatened by Southern Ocean warming.. Proceeding of the National Academy of Science USA.

[36] Deser C, Wallace JM (1987). El Niño events and their relation to the southern oscillation: 1925–1986.. Geophysical Research Letters.

[37] Gregg WW, Conkright ME, Ginoux P, O'Reilly JE, Casey NW (2003). Ocean primary production and climate: Global decadal changes.. Geophysical Research Letters.

[38] Charrassin JB, Bost CA (2001). Utilisation of the oceanic habitat by King Penguins over the annual cycle.. Marine Ecology Progress Series.

[39] Moore JK, Abbott MR (2000). Phytoplankton chlorophyll distributions and primary production in the Southern Ocean.. Journal of Geophysical Research.

[40] Bost CA, Cotté C, Bailleul F, Cherel Y, Charrassin JB (2009). The importance of oceanographic fronts to marine birds and mammals of the southern oceans. Special Issue on Processes at Oceanic Fronts of the Journal of Marine Systems (JMS-SIOF).. Journal of Marine Systems.

[41] Harrell FE (2001). Regression Modeling Strategies: With Applications to Linear Models, Logistic Regression and Survival Analysis..

[42] Martin K (1995). Patterns and mechanisms for age-dependent reproduction and survival in birds.. American Zoologist.

[43] Nisbet ICT, Dann P (2009). Reproductive performance of little penguins Eudyptula minor in relation to year, age, pair-bond duration, breeding date and individual quality.. Journal of Avian Biology.

[44] Corbel H, Morlon F, Geiger S, Groscolas R (2009). State-dependant decisions during the fledging process of king penguin chicks.. Animal Behaviour.

[45] Schmidt-Nielsen K (1984). Scaling: why is animal size so important?.

[46] Gaston A (1997). Mass and date at departure affect the survival of Ancient Murrelet Synthliboramphus antiquus chicks after leaving the colony.. Ibis.

[47] Linden M, Gustaffson L, Pärt T (1992). Selection on fledging mass in the Collared Flycatcher and the Great Tit.. Ecology.

[48] Clutton-Brock TH, Major M, Albon SD, Guinness FE (1987). Early development and population dynamics in red deers. I Density-dependent effects on juvenile survival.. Journal of Animal Ecology.

[49] Tinbergen JM, Boerlijst MC (1990). Nestling weight and survival in individual Great Tits (Parus major).. Journal of Animal Ecology.

[50] Van der Jeugd HP, Larsson K (1998). Pre-breeding survival of barnacle geese Branta leucopsis in relation to fledging characteristics.. Journal of Animal Ecology.

[51] Schwagmeyer PL, Mock DW (2008). Parental provisioning and offspring fitness: size matters.. Animal Behaviour.

[52] Beauplet G, Guinet C (2007). Phenotypic determinants of individual fitness in female fur seals: larger is better.. Proceedings of the Royal Society of London Series B.

[53] Festa-Bianchet M, Gaillard JM, Jorgenson JT (1998). Mass- and Density- dependent reproductive success and reproductive costs in a capital breeder.. American Naturalist.

[54] Lindström J (1999). Early development and fitness in birds and mammals.. Trend in Ecology and Evolution.

[55] Braasch A, Schauroth C, Becker P (2009). Post-fledging body mass as a determinant of juvenile survival in Common Terns hirundo.. Journal of Ornithology.

[56] Danchin E, Boulinier T, Massot M (1998). Conspecific reproductive success and breeding habitat selection implications for the study of coloniality.. Ecology.

[57] Boulinier T, McCoy KD, Yoccoz NG, Gasparini J, Tveraa T (2008). Public information affects breeding dispersal in a colonial bird: kittiwakes cue on neighbours.. Biology Letters.

[58] Viera V, LeBohec C, Côté S, Groscolas R (2006). Massive breeding failures following a tsunami in a colonial seabird.. Polar Biology.

[59] Sousa WP (1984). The role of disturbance in natural communities.. Annual review of ecology and systematics.

[60] Krishnankutty N (2006). Effect of 2004 tsunami on ecosystems – a perspective from the concept of disturbance.. Current Science.

[61] Satheesh S, Wesley SG (2009). Impact of December 26, 2004 tsunami on hydrobiology of Kudankulam coast, Gulf of Mannar, India.. Environmental Monitoring & Assessment.

[62] Mauck RA, Grubb TC (1995). Petrel parents shunt all experimentally increased reproductive costs to their offspring.. Animal Behaviour.

[63] Partridge L, Green P, Sibly RM, Smith RH (1985). Intraspecific feeding specializations and population dynamics.. Behavioural Ecology: Ecological Consequences of Adaptive Behaviour.

[64] Davies N, Green R (1976). The development and ecological significance of feeding techniques in the reed warbler (Acrocephalus scirpaceus).. Animal Behaviour.

[65] Nicolaus M, Le Bohec C, Nolan PM, Gauthier-Clerc M, Le Maho Y (2007). Ornamental colors reveal age in the king penguin.. Polar Biology.

