# Supplementary figures and images for: Effects of Individual Pre-Fledging Traits and Environmental Conditions on Return Patterns in Juvenile King Penguins
Source: PLoS One. 2011 Jun 8;6(6):e20407. doi: 10.1371/journal.pone.0020407 (PMC3110628; doi:10.1371/journal.pone.0020407)

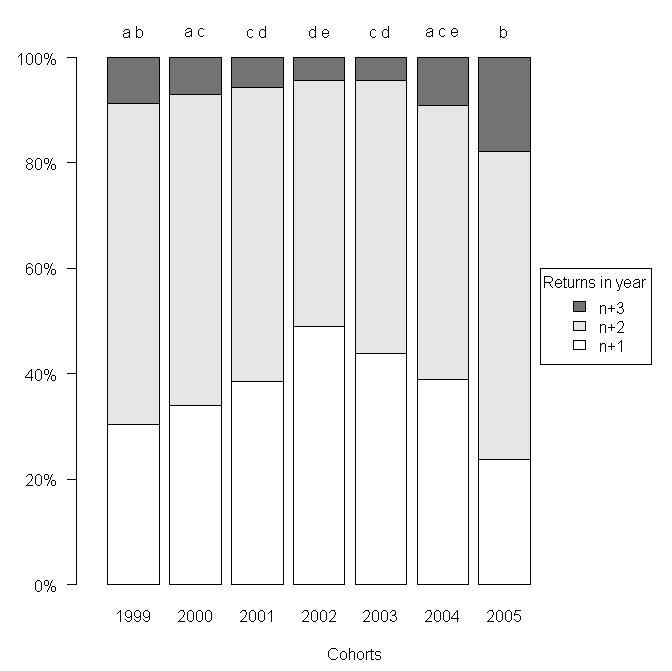

Supplement: Figure S1 — Distribution of the returns of sub-adult king penguins among the 3 years of returns depending on cohorts. Values not sharing a common letter are significantly different. (TIFF) [file pone.0020407.s001.tif]
