# Supplementary material for: Effects of Individual Pre-Fledging Traits and Environmental Conditions on Return Patterns in Juvenile King Penguins
Source: PLoS One. 2011 Jun 8;6(6):e20407. doi: 10.1371/journal.pone.0020407 (PMC3110628; doi:10.1371/journal.pone.0020407)
Supplement: Table S1 — Competitive models tested to explain peak of return inside a return year. (DOC) [file pone.0020407.s002.doc]

**Table S1. Competitive models tested to explain peak of return inside a return year**

| N° | Animal specificity | Year | Depart | Climatic variables | AIC | ΔAIC | w_i_ | k | ED |
| --- | --- | --- | --- | --- | --- | --- | --- | --- | --- |
| P1 | BC + SSI + SEX | Year | Depart | SOI_yret_ + SST_2mret_ | 113.9 | 5.5 | 0.03 | 12 | 0.96 |
| P1.1 | BC + SSI + SEX | Year |  | SOI_yret_ + SST_2mret_ | 112.0 | 3.6 | 0.07 | 11 | 0.96 |
| P1.2 | BC + SSI | Year |  | SOI_yret_ + SST_2mret_ | 109.9 | 1.5 | 0.19 | 10 | 0.96 |
| **P1.3** | **SSI** | **Year** |  | **SOI_yret_ + SST_2mret_** | **108.4** | **0** | **0.41** | **9** | **0.96** |
| P1.4 |  | Year |  | SOI_yret_ + SST_2mret_ | 109.0 | 0.6 | 0.30 | 8 | 0.96 |
| P1.5 |  |  |  | SOI_yret_ + SST_2mret_ | 118.3 | 9.9 | 10^-3^ | 2 | 0.95 |
| P2 | BC + SSI + SEX | Year | Depart | SOI_yret_ + SST_2mfirst_ | 2456.8 |  |  | 12 | 0.03 |
| P3 | BC + SSI + SEX | Year | Depart | SOI_yret_ + SST_wintret_ | 2468.9 | 2360 | <10^-3^ | 12 | 0.02 |

Best model is indicated in bold. ∆AIC is the difference of AIC compared to the best model. w_i_ corresponds to the AIC weight and represents the probability of this model of being the best among the models presented. k is the number of parameters in the model. ED stands for explained deviance and has been calculated as the ratio of the explicated deviance (null deviance – residual deviance) on the null deviance.

BC is the body condition of the animal before departure and SSI is its structural size. Depart is the residual of the regression of BC on the exact date of departure of the bird. SOI_yret_ is the average Southern Oscillation Index on their year of return, SST_2mret_ and SST_wintret_ are the Sea Surface Temperature averaged on the last 2 months and the last winter preceding their return to the colony, respectively.
